# Biofungicidal Potential of *Neosartorya* (*Aspergillus*) *Fischeri* Antifungal Protein NFAP and Novel Synthetic γ-Core Peptides

**DOI:** 10.3389/fmicb.2020.00820

**Published:** 2020-05-13

**Authors:** Liliána Tóth, Györgyi Váradi, Éva Boros, Attila Borics, Hargita Ficze, István Nagy, Gábor K. Tóth, Gábor Rákhely, Florentine Marx, László Galgóczy

**Affiliations:** ^1^Institute of Plant Biology, Biological Research Centre, Szeged, Hungary; ^2^Department of Medical Chemistry, Faculty of Medicine, University of Szeged, Szeged, Hungary; ^3^Institute of Biochemistry, Biological Research Centre, Szeged, Hungary; ^4^Department of Biotechnology, Faculty of Science and Informatics, University of Szeged, Szeged, Hungary; ^5^MTA-SZTE Biomimetic Systems Research Group, University of Szeged, Szeged, Hungary; ^6^Institute of Biophysics, Biological Research Centre, Szeged, Hungary; ^7^Institute of Molecular Biology, Biocenter, Medical University of Innsbruck, Innsbruck, Austria

**Keywords:** *Neosartorya fischeri* antifungal protein, γ-core peptides, biofungicide, cytotoxicity, crop protection

## Abstract

Because of enormous crop losses worldwide due to pesticide-resistant plant pathogenic fungi, there is an increasing demand for the development of novel antifungal strategies in agriculture. Antifungal proteins (APs) and peptides are considered potential biofungicides; however, several factors limit their direct agricultural application, such as the high cost of production, narrow antifungal spectrum, and detrimental effects to plant development and human/animal health. This study evaluated the safety of the application of APs and peptides from the ascomycete *Neosartorya fischeri* as crop preservatives. The full-length *N. fischeri* AP (NFAP) and novel rationally designed γ-core peptide derivatives (PDs) γ^NFAP^-opt and γ^NFAP^-optGZ exhibited efficacy by inhibiting the growth of the agriculturally relevant filamentous ascomycetes *in vitro*. A high positive net charge, however, neither the hydrophilicity nor the primary structure supported the antifungal efficacy of these PDs. Further testing demonstrated that the antifungal activity did not require a conformational change of the β-pleated NFAP or the canonically ordered conformation of the synthetic PDs. Neither hemolysis nor cytotoxicity was observed when the NFAP and γ^NFAP^-opt were applied at antifungally effective concentrations in human cell lines. Similarly, the *Medicago truncatula* plants that served as toxicity model and were grown from seedlings that were treated with NFAP, γ^NFAP^-opt, or γ^NFAP^-optGZ failed to exhibit morphological aberrations, reduction in primary root length, or the number of lateral roots. Crop protection experiments demonstrated that NFAP and associated antifungal active γ-core PDs were able to protect tomato fruits against the postharvest fungal pathogen *Cladosporium herbarum*.

## Introduction

The delicate balance between the demand for food of the increasing world population and global agricultural production is easily disturbed by several different factors that cause enormous crop losses resulting in serious economic and societal impacts (Savary et al., 2012). One of these factors is postharvest plant pathogenic fungi. Fungi threaten the global food supply every year as they are destructive pathogens of agriculturally important plants in fields and spoilage agents of crops during storage (Almeida et al., 2019). Based on the available data from 2010, it is estimated that the amount of the five most important crops (viz., rice, wheat, maize, potato, and soybean) destroyed by fungal infection or contamination that year would be enough to feed 8.5% of the world’s population and would support increasing global calorie consumption for decades (Fisher et al., 2012). In the 20th century, the main strategy for elevating productivity was breeding high yield or fungal pathogen-resistant cultivars in parallel with the application of chemical fungicides in the fields (Savary et al., 2012). Despite these efforts, agricultural fungal damage has steadily been increasing over the last few decades (Fisher et al., 2012). This is likely because fungal pathogens adapt easily to resistant cultivars or quickly develop resistance against novel chemical fungicides (McDonald and Stukenbrock, 2016). Climate change (Elad and Pertot, 2014), global trade, and the transport of agricultural products (Jeger et al., 2011) are facilitating the rapid dispersion of highly virulent and fungicide-resistant plant pathogens or foodborne strains globally. This problem is further exacerbated by the fact that very few new fungicides have been introduced to the global market recently. High research cost, coupled with the rapid emergence of resistant strains, is discouraging investment in the development of new fungicides (Borel, 2017). The application of traditional chemical pesticides in the fields and during storage is still the most common plant and crop protection strategy despite the abovementioned disadvantages (Bardin et al., 2015). Therefore, substantial demand exists for the discovery of novel, cost-effective, and potent fungicides or crop preservatives with a lower risk of developing resistance.

The features of the intensively studied extracellular, small, cysteine-rich, and cationic antifungal proteins (APs) from ascomycetes may meet these requirements and possess potential for utilization as a biofungicide (Leiter et al., 2017). APs exhibit remarkable stability within harsh environmental conditions and are resistant to protease degradation owing to their compact β-fold and disulfide bond-stabilized tertiary structure (Galgóczy et al., 2010). Different APs from *Penicillium* or *Aspergillus* spp. can effectively inhibit the growth of several plant pathogens (Vila et al., 2001; Moreno et al., 2003, 2006; Theis et al., 2005; Barna et al., 2008; Garrigues et al., 2018; Tóth et al., 2020) and foodborne and postharvest mycotoxigenic fungi (Barakat et al., 2010a, b; Delgado et al., 2015, 2017, 2018; Garrigues et al., 2016). Topical application of the *Aspergillus giganteus* AP (AFP) (Moreno et al., 2003) and the *Penicillium chrysogenum* AP (PAF) (Barna et al., 2008) on plant leaves proved to protect *Pelargonium*, barley and wheat, respectively, against fungal pathogens without causing any detrimental effects to the host. Laboratory studies further demonstrated that the AFP (Barakat et al., 2010a) and the *P. chrysogenum* AP (PgAFP) (Delgado et al., 2018) decreased the level of mycotoxin contamination caused by *Fusarium* species and *Aspergillus parasiticus* on stored barley and dry-fermented food products, respectively. Furthermore, expression systems using generally recognized as safe (GRAS) microorganisms, such as *Pichia pastoris* (López-García et al., 2010; Virágh et al., 2014) and *P. chrysogenum* (Sonderegger et al., 2016; Garrigues et al., 2018; Tóth et al., 2018), are already available to produce correctly folded and functional APs in high amounts.

Synthetic antifungally active peptide derivatives (PDs) spanning distinct motifs of the full-length APs are also considered as promising biofungicides. Functional mapping of *Penicillium digitatum* AP B (AfpB) (Garrigues et al., 2017), *Neosartorya (Aspergillus) fischeri* AP 2 (NFAP2) (Tóth et al., 2018), and *P. chrysogenum* PAF (Sonderegger et al., 2018) demonstrated that their synthetic PDs spanning the cationic, surface-exposed loop regions exhibited remarkable *in vitro* antifungal activity. One of these regions is localized at the evolutionary conserved, so-called γ-core motif (GXC-X_[3–9]_-C). This motif is present in extracellular, cysteine-rich antimicrobial peptides and proteins from all biological kingdoms (Yount and Yeaman, 2006), and the amino acid constitution determines the efficacy of the antifungal plant defensins (Sagaram et al., 2011). In our previous study, we introduced a synthetic PD of the native PAF γ-core with anti-*Candida* activity and demonstrated that amino acid substitutions that elevate the positive net charge and hydrophilicity increased the antifungal potency (Sonderegger et al., 2018). And very recently, we described the potential of the *P. chrysogenum* PAF, its designed variant PAF^opt^, and γ-core peptide Pγ^opt^ in plant protection (Tóth et al., 2020).

The present study provides further evidence for the applicability of APs and PDs in agriculture for use as a potential biofungicide or biopreservative agent. We characterized the full-length NFAP from *N.* (*A.*) *fischeri* NRRL 181 and its PD γ^NFAP^ spanning the native NFAP γ-core and used the *de novo* rationally designed variant γ^NFAP^-opt for the *in vitro* antifungal efficacy trial against phytopathogenic ascomycetes. Furthermore, we investigated the physicochemical properties, the structural flexibility influencing the antifungal activity, cytotoxicity on mammalian cell lines and plant seedlings, and their crop protection ability.

## Materials and Methods

### Strains, Cell Lines, and Media

The fungal strains that were tested for antifungal susceptibility are listed in Table 1. They were maintained on potato dextrose agar (PDA, Sigma–Aldrich, St. Louis, MO, United States) slants at 4°C, and susceptibility tests were performed in 10-fold diluted potato dextrose broth (0.1 × PDB, Sigma–Aldrich, St. Louis, MO, United States). Then, the cytotoxicity of NFAP and the PDs was investigated with the following human cell lines THP-1 monocyte cells and HT-29 colonic epithelial cells maintained in RPMI-1640 medium [no 4-(2-hydroxyethyl)-1-piperazineethanesulfonic acid (HEPES), phenol red; Gibco, Thermo Fisher Scientific, Waltham, MA, United States] and HaCaT keratinocyte cells maintained in Dulbecco’s modified Eagle’s medium (DMEM) (high glucose, HEPES, phenol red; Gibco, Thermo Fisher Scientific, Waltham, MA, United States). These media were supplemented with 10% (v/v) fetal bovine serum (Gibco Thermo Fisher Scientific, Waltham, MA, United States) and 1% (v/v) antibiotic/antimycotic solution containing 10,000 U ml^–1^ of penicillin, 10,000 μg ml^–1^ of streptomycin, and 25 μg ml^–1^ of amphotericin B (Gibco, Thermo Fisher Scientific, Waltham, MA, United States). Human cell lines were cultured at 37°C, 5% (v/v) CO_2_ added to the air.

**TABLE 1:** Minimal inhibitory concentrations (μg ml^–1^) of *Neosartorya fischeri* antifungal protein (NFAP), γ^NFAP^, and γ^NFAP^-opt peptides against plant pathogenic filamentous ascomycetes.

Isolate	NFAP	γ ^NFAP^	γ ^NFAP^-opt	Origin of isolate
*Aspergillus flavus* SZMC 3014	100	>200	>200	*Triticum aestivum*/Hungary
*Aspergillus flavus* SZMC 12618	100	>200	>200	*Triticum aestivum*/Hungary
*Aspergillus nige*r SZMC 0145	50	>200	>200	Fruits/Hungary
*Aspergillus niger* SZMC 2759	50	>200	>200	Raisin/Hungary
*Aspergillus welwitschiae* SZMC 21821	25	>200	>200	*Allium cepa*/Hungary
*Aspergillus welwitschiae* SZMC 21832	12.5	>200	>200	*Allium cepa*/Hungary
*Botrytis cinerea* SZMC 21474	50	>200	50	*Fragaria* × *ananassa*/Hungary
*Botrytis cinerea* NCAIM F.00751	50	>200	50	Hungary
*Botrytis pseudocinerea* SZMC 21470	100	>200	100	*Brassica napus*/Hungary
*Botrytis pseudocinerea* SZMC 21471	100	>200	100	*Brassica napus*/Hungary
*Cladosporium herbarum* FSU 1148	100	>200	12.5	n.d.
*Cladosporium herbarum* FSU 969	100	>200	12.5	n.d
*Fusarium boothi* CBS 110250	25	>200	50	*Zea mays*/South Africa
*Fusarium graminearum* SZMC 6236J	25	>200	50	Vegetables/Hungary
*Fusarium oxysporum* SZMC 6237J	25	>200	50	Vegetables/Hungary
*Fusarium solani* CBS 115659	50	>200	12.5	*Solanum tuberosum*/Germany
*Fusarium solani* CBS 119996	100	>200	50	*Daucus carota*/Netherlands

*CBS, Centraalbureau voor Schimmelcultures, Utrecht, Netherlands; FSU, Fungal Reference Centre University of Jena, Jena, Germany; SZMC, Szeged Microbiological Collection, University of Szeged, Szeged, Hungary; NCAIM, National Collection of Agricultural and Industrial Microorganisms, Budapest, Hungary; n.d., data not available.*

### Protein Production and Peptide Synthesis

Recombinant NFAP was produced in *P. chrysogenum* and purified as described previously (Sonderegger et al., 2016). Solid-phase peptide synthesis applying 9-fluorenylmethyloxycarbonyl chemistry was used to generate γ-core PDs of NFAP, according to Sonderegger et al. (2018).

### *In silico* Analyses

Physicochemical properties of the NFAP and γ-core PDs were predicted *in silico*. Molecular weight (Mw), isoelectric point (pI), and the grand average of hydropathy (GRAVY) value were calculated by the ExPASy ProtParam tool (Gasteiger et al., 2005). The total net charge at pH = 7.0 was estimated using the Protein Calculator v3.4 server (The Scripps Research Institute^1^).

### *In vitro* Antifungal Susceptibility Tests

The broth microdilution susceptibility testing method, according to Tóth et al. (2016), was applied to determine the minimal inhibitory concentrations (MICs) of NFAP and the γ-core PDs against phytopathogenic ascomycetes. One hundred microliters of NFAP or γ-core PD solution (0.39–400 μg ml^–1^ in twofold dilutions in 0.1 × PDB) were mixed with 100 μl of 2 × 10^5^ conidia ml^–1^ in 0.1 × PDB in a flat-bottom 96-well microtiter plate (TC Plate 96 Well, Suspension, F; Sarstedt, Nümbrecht, Germany). The medium (0.1 × PDB) without NFAP or the γ-core PDs was added to the conidial suspension to serve as the untreated growth control. Fresh 200 μl 0.1 × PDB was used for the background calibration. The plates were incubated statically for 72 h at 25°C, and then, the absorbance (OD_620_) of each well was measured after shaking for 5 s with a microtiter plate reader operating in well-scanning mode (SPECTROstar Nano, BMG Labtech, Ortenberg, Germany). The absorbance of the untreated control represented 100% growth for the MIC calculation. MIC was defined as the lowest AP/PD concentration at which growth was ≤ 5% in comparison with the untreated control.

Growth percentages for *Cladosporium herbarum* FSU 1148 below the MIC were calculated in comparison with the untreated control to reveal the dose-dependent activity of NFAP and the PDs. In this case, the absorbance of the untreated control culture represented 100% growth for the calculation.

Susceptibility tests were repeated at least two times, including three technical replicates.

### Microscopy

Morphological changes that occurred in the *C. herbarum* FSU 1148 conidia in the presence of NFAP and γ-core PDs were visualized by light microscopy (Axiovert 40 CFL; Zeiss, Oberkochen, Germany) and photographed by a microscope camera (AxioCam ICc 1; Zeiss, Oberkochen, Germany). ZEN 2.3 software (blue edition; Zeiss, Oberkochen, Germany) was used for image processing.

### Electronic Circular Dichroism Spectroscopy

The conformational changes in the structure of NFAP and the γ-core PDs were investigated upon binding to the fungal target cells. Electronic circular dichroism (ECD) spectroscopy application for *C. herbarum* was performed with slight modifications as described previously for *Candida albicans* cells (Kovács et al., 2019). Briefly, *C. herbarum* FSU 1148 conidia were washed three times and suspended in ddH_2_O or an aqueous solution of 100 μg ml^–1^ NFAP or γ-core PD at a final concentration of 10^7^ conidia ml^–1^. ECD spectroscopic measurements of these samples and an aqueous solution of 100 μg ml^–1^ NFAP and PDs were performed in the 185–260 nm wavelength range using a Jasco-J815 spectropolarimeter (JASCO, Tokyo, Japan). Spectra were collected at 25°C with a scan speed of 100 nm s^–1^ using a 0.1-cm path length quartz cuvette. Spectra presented are the accumulations of 10 scans for each sample. Spectrum acquisitions were conducted following 0, 4, and 24 h of incubation of the samples at 25°C and shaking at 210 r min^−1^. Following the spectroscopic measurements, the conidia in the samples were tested for their ability to germinate. To this end, they were washed and suspended in spore buffer [0.9% (m/v) NaCl, 0.01% (v/v) Tween 80], and then, they were streaked in appropriate dilutions (10^5^–10^2^ conidia ml^–1^ in PDB) onto PDA plates in three technical replicates for each sample dilution. The colony-forming units (CFUs) were determined following incubation at 25°C for 72 h. This experiment was repeated twice.

### Cell Toxicity Tests With Human Cell Lines

The potential toxic effects of NFAP and γ-core PDs on the human cell lines were investigated with the application of the CCK8 cell proliferation and cytotoxicity assay kit (Dojindo Molecular Technologies Inc.; Rockville, MD, United States) following the manufacturer’s instructions with slight modifications. Briefly, 20,000 cells per well were preincubated statically in 100 μl (HaCaT and HT-29) or 80 μl (THP-1) of the maintaining medium without phenol red (Gibco, Thermo Fisher Scientific, Waltham, MA, United States) in a flat-bottom 96-well microtiter plate (TC Plate 96 Well, Standard, F; Nümbrecht, Germany) for 24 h in a humidified incubator at 37°C and 5% (v/v) CO_2_ in the air to determine the viability of the cells. Subsequently, the medium from the HaCaT and HT-29 cells was replaced with fresh medium supplemented with NFAP (400–100 μg ml^–1^ in twofold dilution) or γ^NFAP^-opt or γ^NFAP^-optGZ (25–6.25 μg ml^–1^ in twofold dilution). In non-adherent THP-1 cells, 20 μl of NFAP, γ^NFAP^-opt, or γ^NFAP^-optGZ were diluted and then RPMI-640 (without phenol red) was added to reach a final concentration of 50–200 μg ml^–1^ of NFAP or 6.25–25 μg ml^–1^ of γ^NFAP^-opt or γ^NFAP^-optGZ. Then, the plates were incubated for an additional 24 h under the same conditions. Untreated cells (in 100 μl of the respective medium) were used as a viability control, whereas cells treated with 100 μl 50% (v/v) ethanol for only 10 min before measurement served as the dead control. The viability measurement involved the following: the media of adherent HaCaT and HT-29 cells were replaced with the maintaining media without phenol red. Ten microliters of CCK-8 solution was gently mixed by pipetting into each well. Following 2 h the HaCaT and HT-29 or at 4 h THP-1 were incubated at the above-described conditions, the absorbance (OD_405_) was measured using a microplate reader (Hidex Sense Microplate Reader, Turku, Finland). Calculation of cell viabilities included the absorbance of the untreated control, which was assumed to represent 100% growth. Fresh medium without phenol red (100 μl) was used for the background calibration.

The hemolytic potentials of NFAP and the γ-core PDs were tested on Columbia blood agar plates (VWR; Radnor, PA, United States) using disc diffusion as described previously (Sonderegger et al., 2018). Sterile filter discs (Ø 6 mm) were placed on the agar plates, and 10 μl of an aqueous solution of NFAP (4 mg ml^–1^) or γ^NFAP^-opt or γ^NFAP^-optGZ (500 μg ml^–1^) was dropped onto them. Sterile ddH_2_O and 20% (v/v) Triton X-100 were used as negative and positive lysis controls, respectively. The plates were incubated for 24 h at 37°C before the plates were checked for the presence of clear zones around the filter discs.

### Toxicity Tests With Plant Seedlings

*Medicago truncatula*, a fast-growing, small legume, easily cultivable on water agar in Petri dishes (Barker et al., 2006), was an appropriate model organism to investigate the harmful effects of APs and PDs on the growing plants. The NFAP and γ-core PDs were tested for potential toxic effects to the *M. truncatula* A-17 seedlings. Seeds were washed with 96% (v/v) sulfuric acid for 5 min, followed by washing with 0.1% (w/v) mercuric chloride solution for 3 min at room temperature and three times with cold ddH_2_O. Then, the seeds were left to germinate on 1% (w/v) water agar (Agar HP 696; Kalys, Bernin, France) for 3 days at 4°C in the dark. Seedlings with 3–4-mm-long primary roots were selected and transferred to square plates (120 mm × 120 mm × 17 mm Bio-One Square Petri Dishes with Vents, Greiner, Sigma–Aldrich, St. Louis, MO, United States) on fresh 1% (w/v) water agar lining them up at a 20-mm distance from the top. From this line, the bottom part of the plate was covered with aluminum foil to keep the root region in the dark. The apical region of the primary root was treated daily by dripping 20 μl drops of 400 μg ml^–1^ NFAP or 25 μg ml^–1^ γ-core PD in sterile ddH_2_O for 10 days. Plates were incubated in a humid (60%) plant growth chamber at 23°C under continuous illumination (1200 lux). The primary root length was measured, and the number of lateral roots was counted following the treatment period. Sterile ddH_2_O- and 70% (v/v) ethanol-treated seedlings were used as the growth or dead controls, respectively. Toxicity tests were repeated at least two times, and 12 seedlings were involved in each treatment.

### Crop Protection Experiments

The crop protection abilities of NFAP and γ-core PDs were tested on the tomato fruits. The conidial suspension and NFAP and γ-core PD solutions were prepared in 0.1 × PDB. Tomato fruits (“On The Vine Red” variety) were purchased from a local organic farm (Szeged, Hungary). The surface of tomato fruits was sterilized *via* rinsing three times with 20% ethanol (v/v) and then sterile ddH_2_O. Tomato fruits were stung in 3 mm depth at three points near the stalk. Then, (i) infection control, 10 μl of the conidial suspension of *C. herbarum* FSU 1148 (10^7^ conidia ml^–1^), (ii) toxicity testing, 10 μl of the MIC of NFAP or γ-core PDs (100 and 12.5 μg ml^–1^, respectively), (iii) crop protection investigation 10 μl *C. herbarum* FSU 1148 conidial suspension (10^7^ conidia ml^–1^) containing the MIC of NFAP or γ-core PDs, and (iv) for the uninfected control 10 μl 0.1 × PDB were pipetted into the holes, and the treated specimens were then left to dry at room temperature. Unwounded tomato fruits were used as the untreated controls. Incubation included the following: the tomato fruits were kept in a sterile plastic box, humidified with wet paper towels for 7 days at 23°C (average room temperature in shops). Following this incubation period, the tomato fruits were axially cut in half, and then, the presence and depth of the fungal infection were assessed.

### Statistical Analysis

Microsoft Excel 2016 software (Microsoft, Edmond, WA, United States) was used to check the normal distribution of our datasets. Levene’s test (Homogeneity of Variance Calculator-Levene’s Test^2^) was applied to reveal whether the variances of two samples are approximately equal or not. If equal variance was assumed, Microsoft Excel 2016 software was used also to calculate the standard deviations and to determine the significance values (two-sample *t*-test). Significance was defined as *P* ≤ 0.05 based on the following: ^∗^*P* ≤ 0.05, ^∗∗^*P* ≤ 0.005, and ^∗∗∗^*P* ≤ 0.0001. If the two datasets failed in the test of homogeneity, the two-tailed Mann–Whitney *U*-test (Mann–Whitney U Test Calculator^3^) was applied to reveal the significance. Significance was defined as ^∗^*P* < 0.05 in this case.

## Results

### Peptide Design

Physicochemical properties of the NFAP and PDs are summarized in Table 2. The native γ-core motif of NFAP (GECFTKDNTC) is located in the first loop region. It is negatively charged (net charge is −1.1 at pH = 7.0) and slightly hydrophilic (GRAVY = −0.840) (Table 2). The PD γ^NFAP^ spanning the native NFAP γ-core motif was designed based on our recent findings regarding the stability and antifungal efficacy of the γ-core peptides Pγ and Pγ^opt^ from native *P. chrysogenum* PAF (Sonderegger et al., 2018). The synthetic γ^NFAP^ is almost neutral (net charge is −0.1 at pH = 7.0) and hydrophilic (GRAVY = −1.5000) and contains three additional amino acids at the N-terminus and ends in an extra C-terminal lysine residue. The applied N-terminal acetylation and C-terminal amidation mimicked the propagating native protein backbone, neutral terminals, and provided stability against proteolysis. Previously, we observed that cyclization of the Pγ through the disulfide bridge formation impaired antifungal efficacy (Sonderegger et al., 2018); thus, all cysteines in γ^NFAP^ possessed free sulfhydryl (–SH) groups (Table 2). Specific amino acids were substituted in γ^NFAP^ to create the PDγ^NFAP^-opt exhibiting an elevated positive net charge (+5.8 at pH = 7.0) and increased hydrophilicity (GRAVY = −2.264) (Table 2). Then, we investigated whether the net charge or the hydrophilicity influenced the antifungal activity of γ^NFAP^-opt and synthesized two different variants. In the γ^NFAP^-optChZ, amino acid substitutions reduced the net charge from +5.8 to neutral (−0.1 at pH = 7.0) but maintained the GRAVY (Table 2). In contrast, the GRAVY was reduced to −0.557, whereas the net charge remained unchanged (+ 5.8 at pH = 7.0) in the γ^NFAP^-optGZ variant (Table 2).

**TABLE 2:** Amino acid sequence and *in silico* predicted physicochemical properties of mature *Neosartorya fischeri* antifungal protein (NFAP) and peptide derivatives.

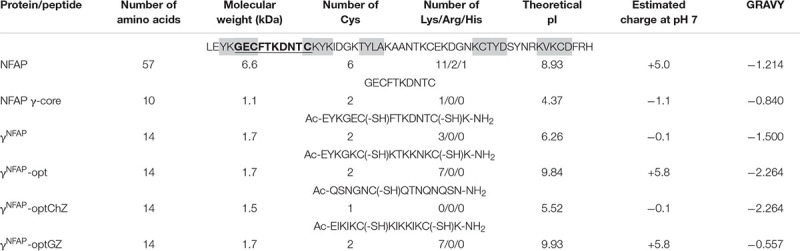

*The γ-core motif in the primary structure of NFAP is indicated in bold and by the underlined letters, the five β-strands are highlighted with a gray background (Hajdu et al., 2019). GRAVY, grand average of hydropathy value; NFAP, *Neosartorya fischeri* antifungal protein.*

### *In vitro* Antifungal Activity of *Neosartorya fischeri* Antifungal Protein and γ-Core Peptide Derivatives on Plant Pathogenic Ascomycetes

The antifungal activity and spectrum of NFAP and the γ-core PDs were investigated in a broth microdilution susceptibility assay. The results are summarized in Table 1. NFAP inhibited the growth of all tested preharvest or postharvest plant pathogenic ascomycetous isolates with various MICs ranging from 12.5 to 100 μg ml^–1^. In contrast, γ^NFAP^ was ineffective (MIC > 200 μg ml^–1^) in the investigated concentration range. The γ^NFAP^-opt effectively inhibited the growth of *Botrytis* (MIC range: 50–100 μg ml^–1^), *Cladosporium* (MIC range: 25–100 μg ml^–1^), and *Fusarium* (MIC range: 12.5–50 μg ml^–1^) isolates; however, aspergilli were not inhibited (MIC > 200 μg ml^–1^).

### Physicochemical Determinants for the Antifungal Efficacy of γ-Core Peptide Derivatives

**FIGURE 1:**
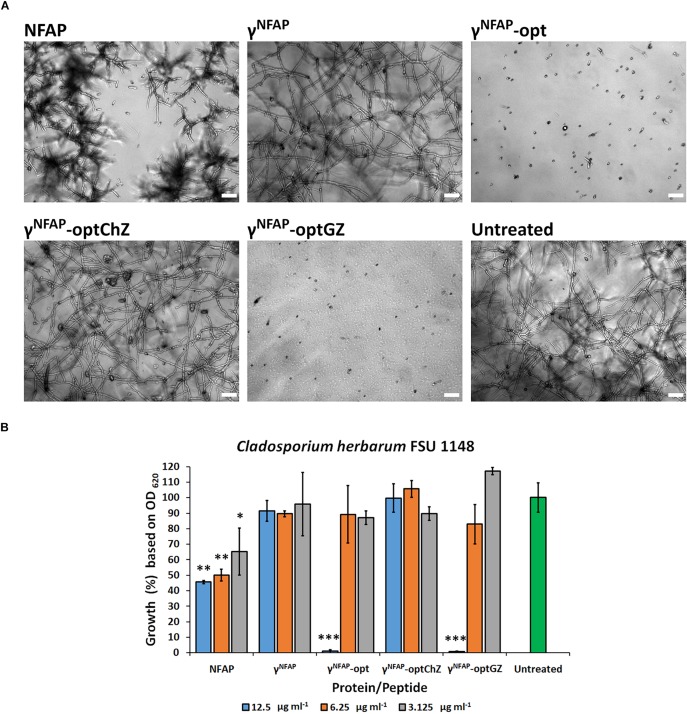
**(A)** Phenotype of the *Cladosporium herbarum* FSU 1148 hyphae evolved from conidia treated with 12.5 μg ml^–1^ *Neosartorya fischeri* antifungal protein (NFAP) and the γ-core peptide derivatives (PDs) γ^NFAP^, γ^NFAP^-opt, γ^NFAP^-optChZ, and γ^NFAP^-optGZ at 25°C for 72 h. Scale bars represent 25 μm. **(B)** Growth percentages of *C. herbarum* FSU 1148 in the presence of different concentrations of NFAP and the γ-core PDs at 25°C for 72 h. Significant differences from two-sample *t*-test in **(B)** are indicated with ^∗^(*P* ≤ 0.05), ^∗∗^(0.0001 < *P* ≤ 0.005), and ^∗∗∗^(*P* ≤ 0.0001) in comparison of each treatment with the untreated control sample.

To understand whether the hydrophilicity or the net charge determines the high antifungal activity of γ^NFAP^-opt, the PD variants γ^NFAP^-optChZ and γ^NFAP^-optGZ (Table 2) were also subjected to antifungal susceptibility test against *C. herbarum* FSU 1148. The neutral and hydrophilic γ^NFAP^-optChZ was inactive at 12.5 μg ml^–1^, which corresponded to the MIC of γ^NFAP^-opt. Fungal growth was visible in the presence of γ^NFAP^-optChZ, and the fungal germlings resembled those of the untreated control (Figure 1A). In contrast, the positively charged but less hydrophilic γ^NFAP^-optGZ inhibited the germination of *C. herbarum* FSU 1148 conidia at the same MIC as γ^NFAP^-opt (12.5 μg ml^–1^) (Figure 1A). These results suggested that the positive net charge, not the hydrophilicity of these γ-core peptides, played a major role in antifungal efficacy. Furthermore, this experiment also indicated that NFAP induced severe morphological changes in the *C. herbarum* hyphae, which resulted in a multibranched phenotype when applied at concentrations below the MIC (12.5 μg ml^–1^). In contrast, γ^NFAP^ did not exhibit any morphological effects on this test fungus at this concentration (Figure 1A). Application of the twofold dilution series of NFAP and the γ-core PDs showed that the full-length protein acts in a dose-dependent manner, whereas the synthetic γ-core peptides γ^NFAP^-opt and γ^NFAP^-optGZ did not show any significant inhibitory effects on the fungal growth below their MIC (Figure 1B).

### The Structure–Function Relation of *Neosartorya fischeri* Antifungal Protein and γ-Core Peptide Derivatives

**FIGURE 2:**
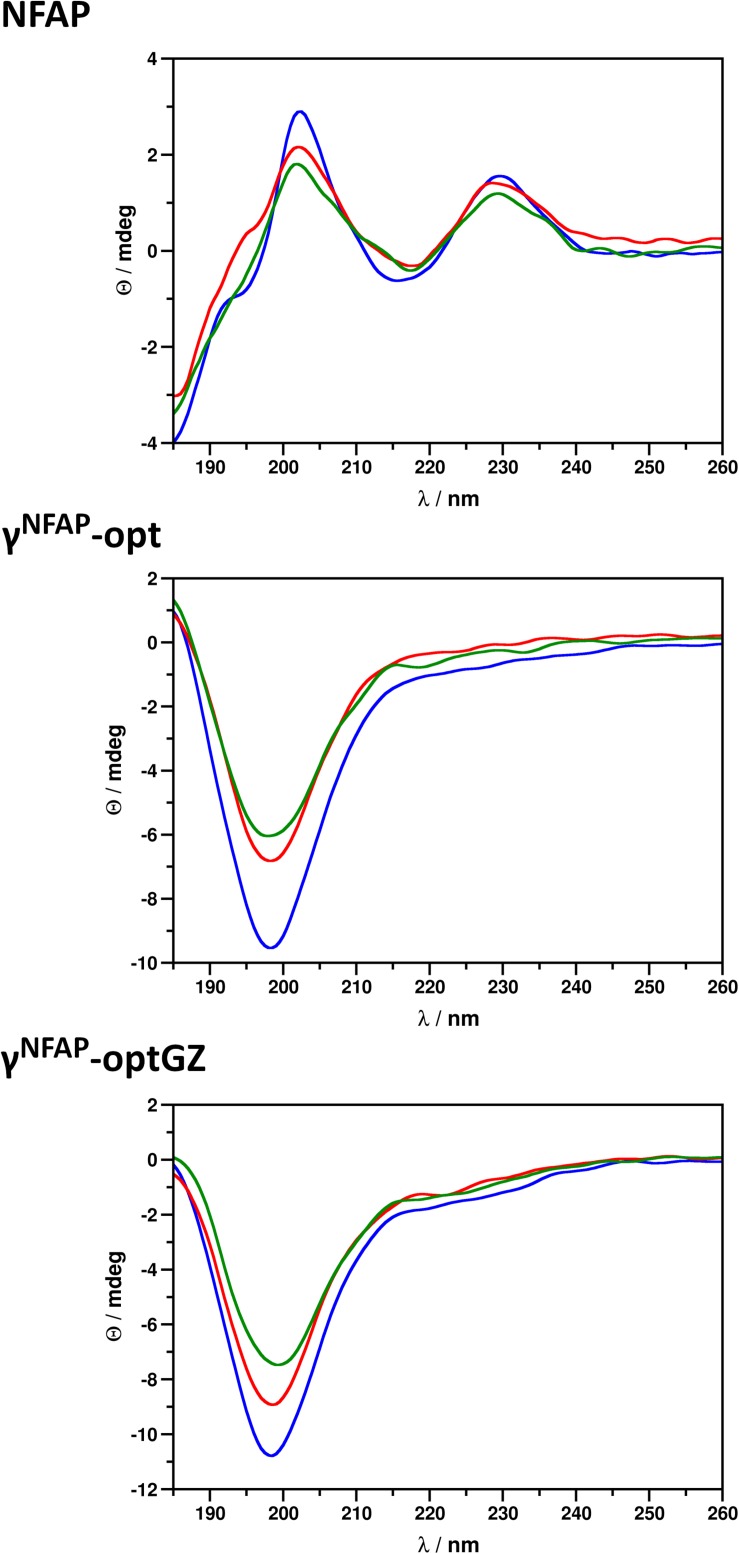
Electronic circular dichroism (ECD) spectra of *Neosartorya fischeri* antifungal protein (NFAP), γ^NFAP^-opt, and γ^NFAP^-optGZ peptides in ddH_2_O (blue) and in the presence of *Cladosporium herbarum* FSU 1148 conidia immediately after exposure (red) to and after 24 h of co-incubation (green) with 100 μg ml^–1^ NFAP, γ^NFAP^-opt, or γ^NFAP^-optGZ.

Several antimicrobially active peptides exerted their effect through the disruption of cell membranes in the sensitive microorganisms. Such mechanisms required extensive conformational changes or peptide oligomerization (Cosentino et al., 2016; Sani and Separovic, 2016; Kumar et al., 2018). ECD spectroscopy was an effective method to monitor such structural changes in the presence of a sensitive microbe (Avitabile et al., 2014). Following 24-h co-incubation of NFAP and *C. herbarum* FSU 1148 conidia did not alter the ECD spectrum of the protein, which indicated that no conformational changes are induced by this interaction with the conidia (Figure 2), whereas the antifungal effect was evident, as indicated by the decreased colony-forming abilities of the conidia following treatment (Table 3). The ECD spectrum of NFAP in the presence of conidia was very similar to that measured previously for pure aqueous solutions of NFAP (Sonderegger et al., 2016), which indicated an intact, disulfide-bridged, β-pleated conformation (Figure 2). Nevertheless, the treatment with 100 μg ml^–1^ NFAP reduced the CFU from 2.2 ± 0.7 × 10^6^ to 1.5 ± 0.7 × 10^6^ (Table 3). Similarly, no conformational changes were observed with any of the antifungal active NFAP γ-core PDs tested. Based on their ECD spectra, γ^NFAP^-opt and γ^NFAP^-optGZ were unordered peptides in aqueous solution (Figure 2). No indication of the ordered structure formation was observed, following 24 h of incubation with *C. herbarum* FSU 1148 conidia (Figure 2). However, again, γ^NFAP^-opt and γ^NFAP^-optGZ significantly reduced the CFU from 2.2 ± 0.7 × 10^6^ to 1.3 ± 0.6 × 10^5^ (*P* = 0.00512) and 0.7 ± 0.6 × 10^5^ (*P* = 0.00512), respectively (Table 3). These results indicated that a canonically ordered conformation is not required for the antifungal effect of the studied peptides. Furthermore, no conformational change could be detected with γ^NFAP^ and γ^NFAP^-optChZ by ECD spectroscopy (data not shown), and expectedly, these PDs did not significantly reduce the ability for colony establishment of *C. herbarum* conidia (Table 3).

**TABLE 3:** Colony-forming unit (CFU) of *Cladosporium herbarum* FSU 1148 conidia treated with 100 μg ml^–1^ *Neosartorya fischeri* antifungal protein (NFAP), γ^NFAP^-opt, and γ^NFAP^-optGZ peptides.

Protein/peptide	CFU (conidia ml^–1^)	*P*-value	Significance
NFAP	1.5 ± 0.4 × 10^6^	0.06576	Ns
γ^NFAP^	2.1 ± 0.4 × 10^6^	0.81034	Ns
γ^NFAP^-opt	1.3 ± 0.6 × 10^5^	0.00512	*
γ^NFAP^-optChZ	2.3 ± 0.4 × 10^6^	0.81034	Ns
γ^NFAP^-optGZ	0.7 ± 0.6 × 10^5^	0.00512	*
Untreated	2.2 ± 0.7 × 10^6^	–	–

Significance was determined in two-tailed Mann–Whitney *U*-test in comparison of each treatment with the untreated control. ns, not significant difference, *P < 0.05.

### Cytotoxic Potential of *Neosartorya fischeri* Antifungal Protein and γ-Core Peptide Derivatives on Human Cell Lines

One of the requirements for new fungicides designed for agricultural applications is their harmlessness in the host. As a proof-of-principle, we tested the cytotoxic potential of NFAP and the two antifungally active γ-core PDs γ^NFAP^-opt and γ^NFAP^-optGZ on human keratinocytes and the colonic epithelial cells. These cell types were in direct contact with APs and PDs if applied as biofungicides, and the treated agricultural products were considered for human consumption. Keratinocytes are the predominant cell type in the epidermis, whereas colonic epithelial cells play a role in nutrient absorption and the innate and adaptive mucosal immunity. Monocytes were also subjected to toxicity tests. They are important parts of the human body’s defense system against infectious organisms and non-self-molecules.

**FIGURE 3:**
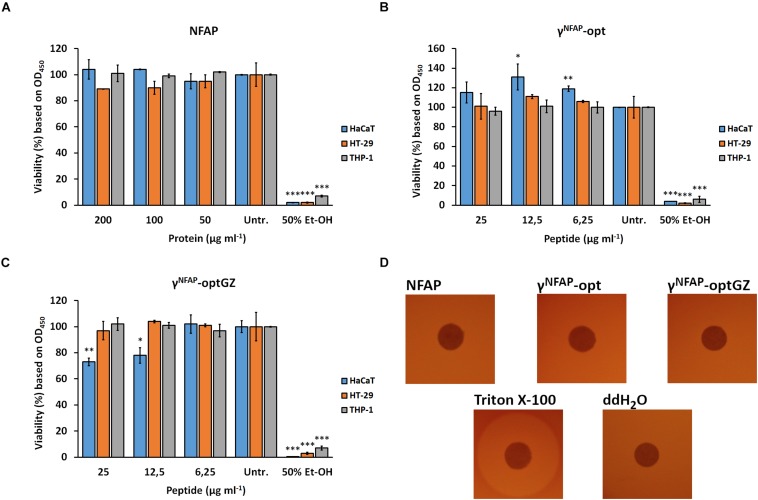
Viability of keratinocytes (HaCaT), colonic epithelial cells (HT-29), and monocytes (THP-1) after treatment with different concentrations of **(A)** *Neosartorya fischeri* antifungal protein (NFAP), **(B)** γ^NFAP^-opt, or **(C)** γ^NFAP^-optGZ peptides for 24 h and with 50% (v/v) ethanol (Et-OH) for 10 min in comparison with the untreated control (Untr.). **(D)** Hemolytic activity of 40 μg NFAP, 25 μg γ^NFAP^-opt and γ^NFAP^-optGZ peptides on Columbia blood agar plates after incubation for 24 h at 37°C. Triton X-100 [20% (v/v)] and ddH_2_O were used as the positive and negative controls, respectively. Significant differences from two-sample *t*-test in **(A**–**C)** are indicated with ^∗^(*P* ≤ 0.05), ^∗∗^(0.0001 < *P* ≤ 0.005), and ^∗∗∗^(*P* ≤ 0.0001) in comparison of each treatment with the untreated control sample.

NFAP and γ^NFAP^-opt did not reduce the viability of the human cell lines in the tested concentration range up to their 2 × MIC (Figures 3A,B). Interestingly, higher viability than the untreated control was detected for the keratinocytes in the presence of 12.5 and 6.25 μg ml^–1^ γ^NFAP^-opt (Figure 3B). In contrast, a significant reduction in the viability of the keratinocytes exposed to 25 and 12.5 μg ml^–1^ γ^NFAP^-optGZ in comparison with the untreated control was observed (Figure 3C). The viability of the other cell lines was not significantly affected by this peptide in the tested concentration range (Figure 3C). The cell membrane disruption ability of NFAP and the antifungally active PDs was investigated on erythrocytes. None of the tested proteins and peptides, NFAP, γ^NFAP^-opt, and γ^NFAP^-optGZ, caused hemolysis (Figure 3D).

### Cytotoxic Potential of *Neosartorya fischeri* Antifungal Protein and γ-Core Peptide Derivatives on Plant Seedling

**FIGURE 4:**
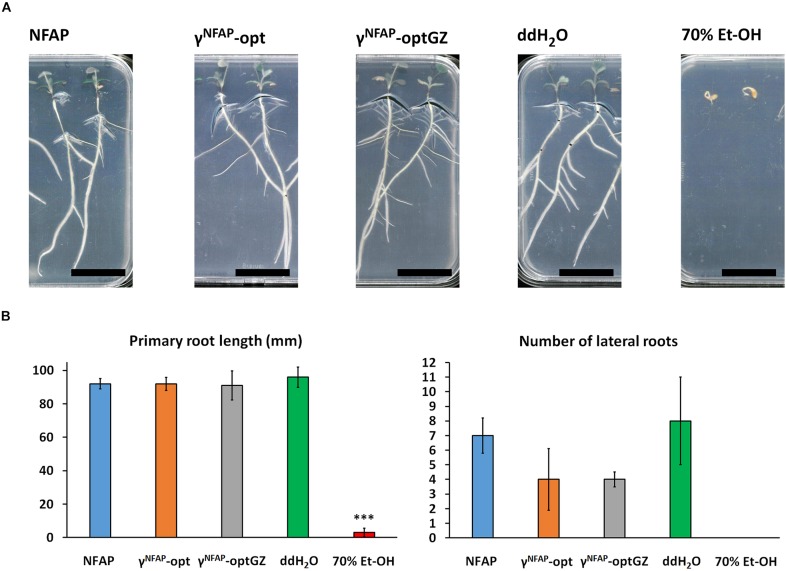
Vegetative growth and development of *Medicaco truncatula* A-17 roots. **(A)** Phenotype of *M. truncatula* A-17 plants grown from seedlings and **(B)** the length of the evolved primary roots and number of lateral roots after treatment with 400 μg ml^–1^ *Neosartorya fischeri* antifungal protein (NFAP), 25 μg ml^–1^ γ^NFAP^-opt, and 25 μg ml^–1^ γ^NFAP^-optGZ peptides for 10 days at 23°C under continuous illumination of the shoot system (1200 lux) in comparison with ddH_2_O- and 70% (v/v) Et-OH-treated controls. Significant difference from two-sample *t*-test in **(B)** is indicated with ^∗∗∗^(*P* ≤ 0.0001) in comparison of each treatment to the ddH_2_O-treated control.

The potential of NFAP and the antifungally active γ-core PDs γ^NFAP^-opt and γ^NFAP^-optGZ to induce morphological aberration and retardation in growing plants was investigated using *M. truncatula* A-17 seedlings. Treatment with 400 μg ml^–1^ NFAP and 25 μg ml^–1^ γ^NFAP^-opt and γ^NFAP^-optGZ did not cause any changes to the plant morphology (Figure 4A). Furthermore, no significant changes in the primary root length and the number of evolved lateral roots were observed following the treatment period (Figure 4B).

### Crop Protection Ability of *Neosartorya fischeri* Antifungal Protein and γ-Core Protein Derivatives

**FIGURE 5:**
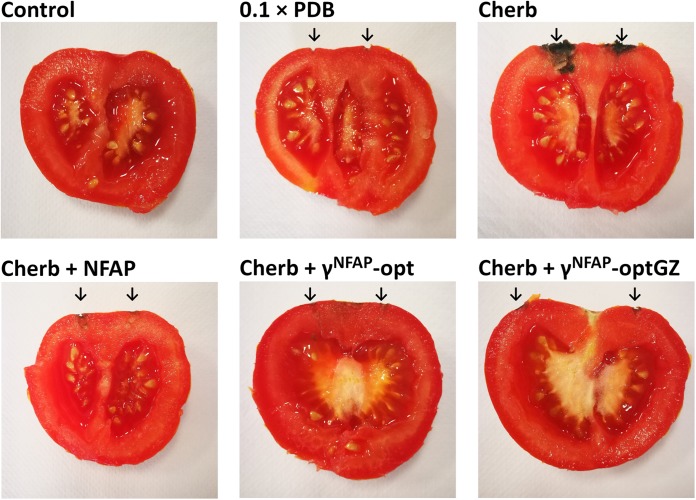
The biopreservation potential of *Neosartorya fischeri* antifungal protein (NFAP) (Cherb + NFAP) and γ-core peptide derivatives (PDs) (Cherb + γ^NFAP^-opt, Cherb + γ^NFAP^-optGZ) of postharvest tomato fruits infected with *Cladosporium herbarum* FSU 1148 after incubation at 23°C for 7 days. The controls were uninfected but treated with medium (0.1 × PDB) or infected with *C. herbarum* FSU 1148 (Cherb) but without AP or PD treatment. Infected tomatoes were treated with 100 μg ml^–1^ NFAP, 12.5 μg ml^–1^ γ^NFAP^-opt, or γ^NFAP^-optGZ, respectively. Unwounded tomato fruits without infection and treatment were used as natural decay controls (Control). The sites of the infections and treatments are indicated with the black arrow.

The ability of NFAP and the antifungally active γ-core PDs to protect crops was studied on tomato fruits against *C. herbarum* FSU 1148. This fungus is known as a postharvest spoilage agent of fresh fruits and vegetables, including tomatoes under storage conditions, especially when the vegetable surface was damaged (Snowden, 1992). Control treatments with NFAP, γ^NFAP^-opt, and γ^NFAP^-optGZ did not cause any decay on the surface of the tomato fruits (data not shown). The same was observed when the fruits were treated with 0.1 × PDB (0.1 × PDB in Figure 5), the medium used for the infection. *C. herbarum* infection, instead, was established within the applied incubation period at the sting points and the deeper tissues (Cherb in Figure 5). Application of NFAP, γ^NFAP^-opt, and γ^NFAP^-optGZ at their MIC inhibited the development of decay. No intensive fungal growth was observed on the surface or in deeper tissues of the tomato fruits (Cherb + NFAP, Cherb + γ^NFAP^-opt, and Cherb + γ^NFAP^-optGZ in Figure 5).

## Discussion

Several APs and antimicrobial peptides from various studies are already in consideration for agricultural application as protective or preservative agents against microbial infections or contamination of plants or stored crops. Their features, such as heat tolerance, a relatively broad antimicrobial spectrum, and low toxicity to plant and mammalian cells, render them to be promising candidates as biopesticides. Primarily, they are applied as recombinant antimicrobial peptides expressed by transgenic plants to confer disease protection (Meng et al., 2010). However, diverse, non-comprehensive international regulations regarding the agricultural application of genetically modified (GM) plants (Tagliabue, 2017) and the prevalence of an anti-GM organism attitude held by policy makers and the general public highly limit the cultivation of these breeds (Lucht, 2015). Furthermore, high production costs, limited information about the antifungal spectrum, the long-term toxic effects regarding plant development and human health are currently obstructing the direct topical application of antifungal peptides and proteins for use as biofungicides or bioprotective agents (Jung and Kang, 2014). The present study provided evidence that AP and the PD applications could be used as a safe and effective topical biofungicide.

The recombinant NFAP produced by the GRAS organism *P. chrysogenum* effectively inhibited the *in vitro* growth of agriculturally important ascomycetes such as *Aspergillus*, *Botrytis*, *Cladosporium*, and *Fusarium* isolates (Table 2). *Aspergillus* and *Fusarium* are well-known plant pathogens and infectious agents of stored crops; they produce mycotoxins that pose a severe threat to both human and animal health (Alshannaq and Yu, 2017). *Cladosporium* spp. are spoilage agents of freshly harvested vegetables and cause significant economic losses every year (De Lucca, 2007). *Botrytis* spp. are aggressive pathogens of the upper parts of numerous plants in the preharvest and postharvest stages (Elad et al., 2007). Recently, several different resistance mechanisms to the chemical fungicides have emerged in the genera *Aspergillus*, *Fusarium* (Lucas et al., 2015; Hawkins et al., 2019), and *Botrytis* (Rupp et al., 2017). NFAP and γ^NFAP^-opt represent potential alternatives to combat chemical fungicide-resistant strains based on our susceptibility test results.

Interestingly, NFAP did not inhibit the germination of *C. herbarum* conidia when it was applied at sublethal concentrations (below the MIC) but affected the hyphal morphology (Figure 1). NFAP induced extensive hyperbranching, similarly to the report on the effect of the morphogenic defensin MsDef1 from *Medicago sativa* (Sagaram et al., 2011) or PAF from *P. chrysogenum* (Kaiserer et al., 2003). We have previously observed this phenomenon with *Aspergillus niger* (Kovács et al., 2011) and *Aspergillus nidulans* (Virágh et al., 2015; Galgóczy et al., 2017) when treated with NFAP. These results evidence that NFAP is a morphogenic AP.

The synthetic γ-core PDs of NFAP, γ^NFAP^-opt, and γ^NFAP^-optGZ (Table 2), with a positive net charge, exhibited potent antifungal activity, whereas the PD γ^NFAP^-optChz with a neutral net charge but the same GRAVY (Table 2) was ineffective against plant pathogenic ascomycetes (Tables 1, 3 and Figure 1). The authors observed a similar correlation between the positive net charge and antifungal efficacy in a previous study with the anti-yeast NFAP2. A PD spanning the neutral NFAP2 γ-core loop region did not inhibit the growth of *Candida* cells. In contrast, a PD derived from the center of the positively charged N-terminal loop region of NFAP2 showed anti-*Candida* activity, whereby the inhibitory potential was independent of the primary structure (Tóth et al., 2018). Another example was reported by Garrigues et al. (2017), who observed that synthetic peptides derived from *P. digitatum* AfpB inhibited the growth of filamentous fungi when they exhibited a high positive net charge. The potential to improve the antifungal efficacy of the peptides by rational design was demonstrated with the peptide Pγ spanning the positively charged *P. chrysogenum* PAF γ-core along with the variant Pγ^opt^. This latter PD inhibited the growth of the *C. albicans* cells more efficiently than the native Pγ because of the elevated positive net charge; however, this occurred independently from the primary structure (Sonderegger et al., 2018). All of these reports so far were evidence that the overall positive net charge was the primary and the most important feature that determined the efficacy of antifungal PDs, irrespective of hydrophilicity, amino acid composition, or primary structure.

Electronic circular dichroism spectroscopy excluded any conformational changes to NFAP to be mandatory for the inhibition of the conidial germination (Figure 2 and Table 3). The same was described before for the potent anti-yeast protein NFAP2 in the presence of the *C. albicans* cells (Kovács et al., 2019). Both proteins shared a similar ECD spectrum, which was characteristic of the small, disulfide-bridged, β-pleated antifungal proteins (Sonderegger et al., 2016; Tóth et al., 2016). We also found that the NFAP γ-core PDs were unordered in the pure aqueous solution, and their antifungal activity was independent of the ordered structure (Figure 2 and Table 3). These observations are parallel with reports of the antifungal activity of the PDs of AfpB (Garrigues et al., 2017) and PAF (Sonderegger et al., 2018) that showed unordered structures under the same experimental setup as the current study utilized. In contrast, membrane disruptive antibacterial peptides underwent a remarkably conformational change when they exerted inhibitory activity on the sensitive bacterial cells (Avitabile et al., 2014). Differences in the mechanistic mode of action between the antifungal and antibacterial peptides or between the structure of the fungal and bacterial cell membrane could explain these contradictions in observations.

The suitability of APs to efficiently protect agriculturally important plants and crops against fungal infection and contamination by direct topical application was proved previously (Moreno et al., 2003; Theis et al., 2005; Barna et al., 2008; Barakat et al., 2010a, b; Barakat, 2014; Tóth et al., 2020). However, the introduction of APs into the biopesticide market requires high ecological compatibility and tolerance by the host (humans, animals, and plants). As for the harmlessness in humans and animals, our proof-of-principle experiments indicated that NFAP and the PD γ^NFAP^-opt were not cytotoxic against keratinocytes, colonic epithelial cells, and monocytes and were not hemolytic in the antifungal effective concentration (Figure 3). Therefore, this *N. fischeri* protein and peptide exhibited harmless activity *in vitro* similar to other members of the fungal APs, such as PAF and AFP (Szappanos et al., 2005, 2006; Tóth et al., 2020), PAF γ-core PDs (Sonderegger et al., 2018; Tóth et al., 2020), and NFAP2 (Kovács et al., 2019). Importantly, experiments with a murine model for pulmonary fungal infection and fungal vulvovaginitis further proved the safety *in vivo* for PAF (Palicz et al., 2013, 2016) and NFAP2 (Kovács et al., 2019), respectively.

So far, no adverse effects of the fungal APs and PDs on plants or fruits have been reported in the literature (Vila et al., 2001; Moreno et al., 2003, 2006; Theis et al., 2005; Barakat, 2014; Garrigues et al., 2018); however, little information is available on the induction of morphological aberrations when growing seedlings with these biomolecules. Exceptions are AFP that did not affect the growth of the tomato plant roots (Theis et al., 2005) or PAF and its derived variant and PD that did neither harm the leaves of barley (Barna et al., 2008) nor those of tomatoes nor affected the seedlings of *M. truncatula* (Tóth et al., 2020). In the present study, the vegetative growth and the morphology of the roots of the *M. truncatula* seedlings were not disturbed by NFAP (Figure 4). We, therefore, suggested that most APs and PDs originating from ascomycetes acted specifically on the fungal cells and were not harmful to the plant cells.

The crop-protective potential of AFP as a topical postharvest preservative has been proven against different *Fusarium* spp. on postharvest barley (Barakat et al., 2010b) and against *Alternaria alternata* on tomatoes, mango fruits (Barakat et al., 2010a), and banana (Barakat, 2014). One important benefit of the AFP treatment was a significant reduction in the mycotoxin burden of the crop (Barakat et al., 2010a). NFAP and the γ-core PDs γ^NFAP^-opt and γ^NFAP^-optGZ similarly proved effective as biopreservatives for tomato fruits as they prevented the contamination by the postharvest mold *C. herbarum* (Figure 5) equally, which suggested a promising novel compound for the prevention of mycotoxin contamination in food products.

## Conclusion

Taken together, our study demonstrated that NFAP and rationally designed synthetic PD γ^NFAP^-opt are promising candidates for biopreservation in agriculture and food industry. However, further studies that focus on their environmental impact and address their pharmacokinetic properties in the human body are essential to push forward their applicability.

## Data Availability Statement

The datasets generated for this study are available on request to the corresponding author.

## Author Contributions

IN, GT, GR, FM, and LG conceived and supervised the study, designed the experiments, and edited the manuscript. GV, FM, and LG performed peptide design. GV performed peptide synthesis. LT and HF performed protein preparation, *in vitro* antifungal susceptibility tests, and plant toxicity assay and analyzed the related data. AB and HF performed ECD spectroscopy and analysis of the related data. LT and ÉB performed cell viability assay and analyzed the related data. LT and LG performed crop protection experiments and analysis of the related data. LT, AB, IN, GT, GR, FM, and LG wrote the manuscript and made manuscript revisions. All authors read and approved the submitted version.

## Conflict of Interest

The authors declare that the research was conducted in the absence of any commercial or financial relationships that could be construed as a potential conflict of interest.

## Footnotes

1 http://protcalc.sourceforge.net

2 https://www.socscistatistics.com/tests/levene/default.aspx

3 https://www.socscistatistics.com/tests/mannwhitney/default2.aspx
